# Temporally Evolving and Context-Dependent Functions of Cytokines That Regulate Murine Anti-*Plasmodium* Humoral Immunity

**DOI:** 10.3390/pathogens11050523

**Published:** 2022-04-29

**Authors:** Fionna A. Surette, Noah S. Butler

**Affiliations:** Department of Microbiology and Immunology, Immunology Graduate Program, University of Iowa, Iowa City, IA 52242, USA; fionna-surette@uiowa.edu

**Keywords:** interleukin-10, interleukin-21, type I interferon, germinal center, T follicular helper, CD4 T cell, B cell, *Plasmodium*, malaria

## Abstract

Protective immunity against blood-stage *Plasmodium* infection and the disease malaria depends on antibodies secreted from high-affinity B cells selected during the germinal center (GC) response. The induction and stability of the GC response require the activation and direct cell–cell communication between parasite-specific CD4 helper T cells and B cells. However, cytokines secreted by helper T cells, B cells, and multiple other innate and adaptive immune cells also contribute to regulating the magnitude and protective functions of GC-dependent humoral immune responses. Here, we briefly review emerging data supporting the finding that specific cytokines can exhibit temporally distinct and context-dependent influences on the induction and maintenance of antimalarial humoral immunity.

## 1. Introduction

Protozoan *Plasmodium* parasite infections are responsible for >240 million clinical cases of malaria and >620,000 deaths annually [1]. Transmission of *Plasmodium* parasites is facilitated by their vector, female *Anopheles* mosquitos. Sporozoites develop from oocysts in the mosquito gut and are transported in hemolymph to salivary glands where they are subsequently released into the host dermis during a blood meal. Sporozoites traffic from the dermis to the bloodstream eventually reaching the liver where they infect hepatocytes and differentiate into merozoites. The release of merozoites from infected hepatocytes results in their rapid attachment to, and invasion of, host red blood cells (RBCs) [2,3]. Within RBCs, merozoites undergo asexual reproduction and continue with the cyclical invasion of RBCs and subsequent amplification of infection [4]. Parasite-infected RBCs adhere to inflamed endothelium with subsequent sequestration of infected RBCs in vital organs such as the brain. These processes, termed cerebral malaria, disrupt the microvasculature causing seizures, stroke, and death [5].

Infected RBC clearance and prevention of cytoadherence require the infected host to mount antibody responses directed against key parasite proteins expressed on the surface of infected RBCs. However, anti-*Plasmodium* humoral immunity and parasite-specific secreted antibody responses are short-lived and non-sterilizing. Weak and ineffective humoral immunity directly contribute to recurrent *Plasmodium* infections in individuals in endemic areas, sustaining malaria transmission and highlighting the need for a deeper understanding of the mechanisms that regulate these processes. In addition to major histocompatibility class II (MHCII) and T cell receptor (TCR) engagement and costimulatory molecule signaling, the differentiation and functional capacity of immune cells are shaped by the unique cytokine milieu that is induced by *Plasmodium* infection. In both humans and experimentally infected rodents, malaria triggers the expression of a balance of pro-and anti-inflammatory cytokines that are important for both mediating an antiparasitic response and limiting host immunopathology. While correlations between cytokine profiles and disease states can be made in humans, experimental systems are necessary to dissect mechanisms through manipulation, and as such, this review focuses on murine *Plasmodium* infection. Knockout mouse models have historically provided a context to study the influence of specific cytokines on initiating and regulating host immune responses, though such studies are limited in their ability to examine the roles of cytokines in ongoing and resolving responses. Models that allow for transient modification of cytokines will be important to dissecting the temporally evolving and context-dependent functions of cytokines reported to be critical for orchestrating anti-*Plasmodium* immunity.

## 2. GC Initiation

Generating effective germinal centers is critical for high-affinity humoral immunity to pathogens. At homeostasis, B cell follicles within secondary lymphoid organs (SLO) are populated by naïve B cells and a network of follicular dendritic cells (FDC). B cell follicles are separated from T cell zones by the T–B border, while follicles themselves are separated by the interfollicular zone. Chemotactic factors expressed by cells within their respective compartments within the SLO are important for establishing gradients that promote spatial separation of these areas at homeostasis, as well as the directed movement of lymphocytes through these compartments upon infection and germinal center formation. The chemokine receptor CXCR5 is expressed by B cells and directs their homing toward follicular stromal cells, such as FDCs and marginal reticular cells (MRCs), that express comparatively higher concentrations of the ligand, CXCL13. By comparison, naïve T cells expressing the chemokine receptor CCR7 are drawn toward high concentrations of its ligands, CCL19/21, expressed by stromal cells within T cell zones but not follicles. These gradients of CXCL13 and CCL19/21 function to establish lymphoid follicles and T cell zones, respectively [6,7]. The expression of the Epstein–Barr virus-induced G-protein-coupled receptor 2 (EBI2, or GPR183) is also important for the localization of splenic DCs in marginal zone bridging channels, as well as for B cell positioning within follicles [8,9,10,11]. The primary ligand of EBI2, 7α,25-dihydroxycholesterol (7α,25OHC), is a metabolic product of stromal cells concentrated at T–B borders and interfollicular regions within the spleen.

Following infection or vaccination, dendritic cells (DCs) and B cells act cooperatively to induce and reinforce the differentiation of CD4^+^ T follicular helper (Tfh) cells. In an initial DC priming phase, TCR engagement, ICOS–ICOSL [12] interaction, and activated DC-derived interleukin (IL)-6 drive STAT1/STAT3-mediated [13] upregulation of the transcriptional regulator Bcl-6 [14] in naïve CD4^+^ T cells. Tfh commitment is further promoted via rapid expression of IL2Rα (CD25) by DCs that promotes quenching of IL-2 [15], thereby limiting STAT5 activation in CD4^+^ T cells and the expression of the transcriptional repressor Blimp-1, an antagonist of Bcl-6. Tfh cells subsequently upregulate CXCR5 [16] and EBI2 [15] and downregulate CCR7 [17], which directs the movement of activated CD4^+^ T cells away from high concentrations of CCL19/CCL21 within the T cell zone and toward the CXCL13 and 7α,25OHC rich regions of the T–B border or interfollicular zone. ICOS–ICOSL interactions between activated CD4^+^ T cells and non-cognate B cells induce pseudopod formation on CD4^+^ T cells that further promotes movement toward the T–B border [18]. Cognate, antigen-specific interactions between Tfh cells and B cells reinforce Bcl-6 and repress EBI2 expression, as well as further upregulate CXCR5 in Tfh cells, promoting the movement of both B and Tfh cells to the follicle center to initiate GC formation [11,19,20,21]. Although chemokines and metabolites play critical roles in governing these dynamic events during homeostasis and GC formation, multiple cytokines have recently been implicated in regulating key aspects of the initiation and reinforcement of protective humoral immune responses during *Plasmodium* infection, including IL-10, IL-21, and type I IFN.

### 2.1. Interleukin-10

Studies in IL-10 knockout mouse models have established that IL-10 plays a critical role in the resolution of *Plasmodium* infections. In the absence of IL-10, *P. chabaudi chabaudi* (*Pcc*)-infected mice exhibit a reduction in survival preceded by an increase in IFN-γ and TNFα [22] production during the first week of infection, as well as increased edema and hemorrhages in the brain by day 8 p.i. [23]. IL-10-deficient mice also uniformly succumb to infection with the *P. yoelii* (*Py*) 17XNL line [24]. In this model, B cells respond directly to IL-10, which appears to counteract the effects of IFN-γ and promote the accumulation of GC B cells [24]. DC-derived IL-10 acts to limit the early (day 5) production of DC- and CD4^+^ T cell-derived IFN-γ, as well as DC-derived TNFα [25]. In the lethal *P. berghei* ANKA (*Pb*A) infection model, administration of recombinant IL-10 during infection prolongs survival [26]. While these studies establish the critical importance of IL-10, they neither directly investigated the mechanisms by which IL-10 promotes GC B cell responses nor revealed whether IL-10 functions during a defined interval to promote survival and anti- *Plasmodium* immunity.

More recently, several groups have examined the temporal impacts of IL-10 during *Plasmodium* infection. Using the *Py* 17XNL model of infection, IL-10 was recently shown to act within the first 4 days p.i. to promote humoral immunity. When IL-10 is blocked during the first 4 days of infection, mice have increased parasitemia and succumb to infection, while no such phenotypes are observed if IL-10 is blocked after day 4 p.i. [27]. Although IL-10 did not influence the control of acute *Py* 17XNL after day 4 p.i., this cytokine is still produced at later time points and can exert function in other contexts. For example, in a co-infection model of *Py* and *Salmonella typhimurium* (*St*), *Py* line N67 infection exacerbates *St* burden, an effect that is lessened by blocking IL-10 on days 7–11 post-*Py* infection [28]. Notably, control of *St* burden is directly mediated by IFN-γ, which is itself limited by IL-10, so this cross-regulation may account for the protective role IL-10 plays at a later time point in this context [29]. In lethal models of *Plasmodium*, IL-10 appears to play differing roles. Following lethal *Py* line 17XL infection, IL-10 appears to limit survival, as there is an increase in survival following neutralization of IL-10 beginning on day 2 p.i. [30] By contrast, administration of recombinant IL-10 to *Pb*A-infected mice on days 3–10 p.i. prolongs host survival [26]. IL-10 also regulates clinical symptoms and survival in *Pcc-*immune mice that were subsequently challenged with lethal *Pb*A. *Pcc*-immune mice exhibited a 25% reduction in survival and increased clinical score when an anti-IL-10 receptor antibody was administered throughout secondary *Pb*A infection, while no such differences in these clinical parameters were observed following primary *Pb*A infection and IL-10 signaling blockade [31]. In *Pcc*-immune mice treated with isotype control antibody and challenged with *Pb*A, substantial increases in activated CD11a^hi^CD49d^hi^ CD4^+^ T cells, IFN-γ producing CD4^+^ T cells, γδ T cells, and B cells were observed [31], suggesting that IL-10 plays a role in suppressing the early expansion of memory CD4^+^ T cells, as well as the expansion of γδ T cells, which have recently been appreciated to phagocytose infected erythrocytes during *Plasmodium* infection [32,33].

Regarding the mechanisms by which IL-10 bolsters humoral immunity, recent data show that IL-10 promotes B cell activation and expression of MHCII and adhesion molecules, while also limiting IFN-γ induced immunopathology [27]. Kinetics of IFN-γ expression varies during lethal versus non-lethal experimental *Plasmodium* infections, which may influence both the timing and mechanisms by which IL-10 exerts protective functions. During a co-infection model with *St*, where IFN-γ contributes to survival and IFN-γ^+^ CD4^+^ T cells do not reach their numerical peak until the third week of infection, IL-10 is detrimental in the second week of infection [34,35]. Similarly, IL-10 may broadly act to aid in the induction of humoral immunity early during *Plasmodium* infection, while its role at later time points is context-dependent.

### 2.2. Interleukin-21

IL-21 is a pleiotropic cytokine that functions to regulate innate and adaptive immune responses and the cellular functions of NK cells, DC, T cells, and B cells. T cell-derived IL-21 acts on B cells to promote the development of GC B cells [36] and restrict the development of early antigen-specific CD38^hi^ memory-like B cells [37]. IL-21 has additionally been shown to promote Bcl-6 expression in GC B cells, both alone [38] and in conjunction with IL-4 [39]. While IL-21 promotes GC B cell development, GCs remain visible by microscopy in the absence of IL-21 or IL21R, and no change in Tfh cell differentiation occurs [37]. T cell-derived IL-21 has been shown to act on B cells during *Pcc* infection to promote control of chronic parasitemia and the formation of antigen-specific MBC populations in the spleen, as well as long-lived, bone marrow B220^+^CD138^hi^ plasmablasts [36]. These differ from short-lived, extrafollicular plasmablasts, which when numerically over-represented early after *Plasmodium* infection (day 10 p.i.) appear to constrain GC-dependent humoral immunity [40].

T-bet^+^Bcl-6^+^ T helper type 1 Tfh cells (Tfh1) capable of producing both IL-21 and IFN-γ have been observed in multiple disease states [41,42,43,44]. Following *Pcc* infection, these dual, cytokine-producing Tfh1 cells are the dominant IL-21 producing CD4^+^ T cell subset [36]. During the DC priming phase, activated DC-derived IL-12 may contribute to the appearance of Tfh1 cells, as human naïve CD4^+^ T cells cultured with activated DCs produce IFN-γ and IL-21, an effect that is lost when IL-12 is blocked [43]. In line with this, human naïve CD4^+^ T cells treated with IL-12 differentiate into T-bet^+^Bcl-6^+^ Tfh1 cells that are capable of producing IL-21^+^ and IFN-γ^+^ in a STAT4-dependent manner [43,44]. Tfh1 cells may additionally differentiate from Th1 cells; in vitro, Th1 polarized primary mouse lymphocytes are capable of further differentiation into Tfh1 cells that dually express T-bet and Bcl-6 in the presence of IL-12 and low IL-2, which is regulated in a STAT3- and STAT4-dependent manner [45]. The specific cytokine milieu is important for these fate decisions, as higher concentrations of IL-2 favor differentiation of Th1 over Tfh1-like cells in vitro [45].

Notably, few studies have examined the role of IL-21 outside the context of immune response initiation during parasitic infections. Similar to Il21^−\−^ studies, blockade of IL-21R from days 4–9 after infection with *P. berghei* line XAT, a low virulence strain of *P. berghei*, results in a significant decrease in GC B cells, though GC B cells are still detectable by flow cytometry [46]. Without the inclusion of Bcl-6 staining or imaging for localization, it is difficult to interpret how the absence of IL-21 following initial B cell activation impacts subsequent GC colonization and antibody quality. In contrast to what is known about the role of IL-21 in GC B differentiation, during *Litomosoides sigmodontis* infection coupled with IL-21R blockade on days 45–60 p.i., there is an increase in the frequency of PNA^+^ B cells and parasite-specific IgG1 at day 60 p.i., correlating with a decrease in larval microfilaria [47]. Similar to what is observed upon GC initiation, there are no changes in Tfh cells following IL-21 neutralization in this model [47]. In the limited research examining the temporal role of IL-21 during parasitic infections, this cytokine appears to play opposing roles in supporting antibody production at early and late time points post-infection.

### 2.3. Type I Interferons (IFN-α/β)

Utilizing knockout mice or antibody-mediated receptor blockade, several context-dependent roles for type I IFN in *Plasmodium* infection have been reported, with most experiments showing type I IFN primarily acting to promote malaria-associated pathology. IFN-α contributes to increased parasitemia, brain hemorrhages, and increased clinical scores in *Pb*A-infected C57BL/6 mice [48]. IFNαR1^−\−^ mice exhibit decreased whole body parasite burden that may be partially mediated by brain and liver infiltrating IFN- γ^+^ CD4^+^ T cells [48], as this protective phenotype is lost when CD4 T cells are depleted. During blood-stage *Py* infection, type I IFN signaling similarly contributes to increased parasitemia [49]. T cell-intrinsic type I IFN signaling partially promotes the accumulation of Blimp-1^+^ T-bet^+^ IFN-γ and IL-10 co-expressing T regulatory 1 (Tr1) cells whose cytokine secretion appears to indirectly limit humoral immunity [49]. In line with this, IFN-α signaling promotes IFN-γ and IL-10 production from human PBMC-derived CD4+ T cells isolated following *P. falciparum* infection [50]. Additionally, IFN-α suppresses IL-6, IL-1β, and IL-17 but not TGF-β, up to 4 weeks post-infection, suggestive of an environment supportive of Tr1 differentiation [50].

Splenic red pulp macrophages, plasmacytoid DCs, and cDCs produce IFN-β during *Plasmodium* infections, although control of parasitemia does not rely on red pulp macrophages [51] and only partially relies on pDCs [52,53]. Bone-marrow-derived pDCs produce IFN-β as early as 24 h post-*Py* YM infection, a process that is reliant on CD169^+^ macrophages in the bone marrow and blood [53]. pDCs and CD169^+^ macrophages form long-lasting interactions by 36 h post-*Py* YM infection, although the increase in IFN-β as early as 24 h post infections suggest they may interact earlier, or there are additional sources of IFN-β at this early time point [53]. IFN-α and IFN-β are produced throughout *Plasmodium* infection; while expression peaks early post-infection, their contribution at later time points has only been assessed up to 5 days p.i.

In the context of *Pb*A infection, IFNαR1^−\−^ mice are fully protected from ECM, while WT C57BL/6J mice exhibit nearly 100% incidence of ECM. However, blocking IFNαR1 signaling on days 5–7 p.i. results in only partial protection from ECM, with ~30% of treated mice succumbing to ECM [54]. Others have shown a protective effect of type I IFN. *Pb*A-infected mice treated with recombinant human IFN-α on days 0–25 p.i. maintain low blood parasitemia [55]. Mice treated on days 3–12 p.i. maintain low parasitemia initially, and while blood parasitemia increases by day 16 p.i., both groups were protected from cerebral malaria, compared with controls [55]. Following *Py* YM infection, recombinant IFN-α/β fully protects mice from death when administered i.v. 18 h p.i. [52]. IFN-α/β administration at 32 h p.i. partially protects mice from death and parasitemia, while administration at 48 h affords no protection, compared with controls [52]. A separate group investigating type I IFN during *Py* YM infection found that IFN-α expression peaks on day 2 p.i. and contributes to mortality, as infected IFNαR1^−\−^ mice survive at least twice as long as WT controls and IFNαR1^+\−^ mice [53]. Future research is required to address the discrepancies between these studies in the context of *Py* YM infection. Despite conflicting conclusions on the pathogenic or protective role of type I IFN during lethal *Plasmodium* infection, they appear to exert function early post-infection, with later treatment resulting in diminished protective or pathogenic effects.

### 2.4. Influence of Type I Interferons on Generation of Tfh1 and Atypical Memory B Cells (atMBCs)

IL-21^+^ and IFN-γ^+^ Tfh1 cells have been observed at both acute [36] and memory [56] time points following *Pcc* infection; however, without selective deletion or transfer of this population, it is difficult to say whether their function changes over time, compared with IL-21^+^ Tfh. As the development of atMBC appears partially dependent on IL-21^+^ and IFN-γ^+^ [42], and Tfh1 cells themselves are partially dependent on type I interferons [57], addressing the function of Tfh1 during *Plasmodium* infection is of particular interest. atMBCs are commonly described by expression of CD11c, T-bet, or FCRL5 in mice [58], or additionally CXCR3^+^CD27^−^ CD21^-^ in humans, and have been associated with age, viral, bacterial, or parasite infection, as well as autoimmune and metabolic diseases [59]. atMBCs correlate with anemia and autoantibodies during *P. falciparum* and *P. vivax* infections in humans [60,61], and even though they are found in uninfected humans, they are expanded up to 16% in individuals living in malaria-endemic regions [62]. In a mouse model of lupus, type I interferons have been shown to partially support the expansion of Tfh1 cells [57]. T cell help appears to support atMBC expansion early, as CD40L blockade on days 16–30 p.i. with *Ehrlichia muris* results in a nearly threefold reduction in atMBC cells, while blockade on days 30–37 p.i. results in a less drastic reduction, though this may additionally relate to the duration of the blockades [42]. Interestingly, during murine viral infection, atypical B cells (atB) appear to localize outside of GCs, clustering at the marginal zone, and be non-GC derived, supporting the idea that atB formation occurs early post-infection [63]. As atMBCs survive to memory time points and expand upon the *Plasmodium* challenge, it will be important to dissect the temporal role that IL-21^+^ and IFN-γ^+^ Tfh1 cells and type I interferons have on atMBC development and function during *Plasmodium* infection.

### 2.5. Outstanding Questions

IL-10, IL-21, and type I IFNs are crucial for mounting efficient immune responses against *Plasmodium* parasites and protecting hosts from lethal immunopathology. Future studies examining the temporal and context-dependent role of cytokines on the initiation, maintenance, and dissolution of GCs and the formation and function of memory B and CD4^+^ T cells are warranted. This is highlighted by findings revealing the short time frame in which IL-10 appears to act during acute *Plasmodium* infection, as well as studies showing a striking difference in how IL-21 regulates GC initiation versus memory B cell formation following *Litomosoides sigmodontis* infection. As *Plasmodium*-infected humans are likely to have been previously exposed and have received interventional treatment for previous infections that blunt GC reactions, it will be important to address if IL-21 influences the formation of memory populations when concentrations are preemptively moderated. An additional and critical question relates to whether memory populations that form under these conditions functionally respond to the challenge. Moreover, Tfh1, atMBC, and Tr1 cells have been well described during *Plasmodium* infection. Thus, whether the altered IL-10 or IL-21 expression at later or chronic stages of infection influences the likelihood of memory populations to adopt the phenotype of atypical B cells or Tr1 cells, and whether this impacts functional memory, remain priority questions. Despite recent data establishing the critical importance of these cytokines in regulating the GC response, several fundamental knowledge gaps remain regarding the potential for temporally evolving roles in regulating humoral immunity in response to either infection or vaccination.

## Data Availability

Not applicable.
